# Assessing Impacts of Climate Change on Phenology and Quality Traits of *Vitis vinifera* L.: The Contribution of Local Knowledge

**DOI:** 10.3390/plants8050121

**Published:** 2019-05-09

**Authors:** Rita Biasi, Elena Brunori, Carlotta Ferrara, Luca Salvati

**Affiliations:** 1 Department for Innovation in Biological, Agrofood and Forest systems (DIBAF), University of Tuscia, via S. Camillo de Lellis, snc, 01100 Viterbo, Italy; brunori@unitus.it; 2 Research Centre for Forestry and Wood, Viale Santa Margherita 80, 52100 Arezzo, Italy; carlotta.ferrara@crea.gov.it (C.F.); luca.salvati@crea.gov.it (L.S.)

**Keywords:** bioclimatic indices, biotic stress, berry ripening, resilient viticulture, vineyard micro-climate

## Abstract

Developing adaptation strategies in *Vitis vinifera*, a crop sensitive to climate change, is crucial for resilience of traditional viticultural systems, especially in climate-vulnerable areas like the Mediterranean basin. A progressive warming is demonstrated to alter the geographical distribution of grapevine, reducing land capability for typical grapes and vine productions in most Southern European districts traditionally specialized in tree crops. Grapevine growth and reproduction under climate change require a continuous monitoring to adapt agronomic practices and strategies to global change. The present study illustrates an empirical approach grounded on a set of bio-physical indicators assessing the genotype-related response to climate variation. This approach was tested in Umbria, central Italy, to verify the response of some major international and local grapevine varieties to climate variation during a relatively long time interval (1995–2015). Long-term data for ripening time and berry quality collected in the study area were correlated to representative bioclimatic indices including Winkler, Huglin, and Cool night indicators. Results of this study highlighted the increase of air temperature (reflecting the inherent growth in thermal availability for maturation) and the alteration of precipitation patterns toward more intense precipitation. Climate variability exerted distinctive impacts on grapevine phenology depending on the related genotype. Empirical findings underline the usefulness of a permanent field monitoring of the relationship between selected climate variables and grape ripening with the aim to develop adaptive viticultural practices at farm’s scale.

## 1. Introduction

Climate, a main component of the ‘terroir’ notion, is a key factor for grapevine geographical distribution across the world [1,2], as well as for grape and wine quality [3,4], and healthiness [5]. Thanks to the high plasticity to environmental shocks, grapevine cultivation is diffused all over the world, adapting to different physiographic conditions as far as latitude and elevation are concerned (www.OIV.org). Nonetheless, grapevine performances are influenced by climate variability—primarily by thermal availability—and this species has been increasingly recognized as a bio-indicator of global warming [6]. Climate adaptation strategies are especially required to minimize climate change impact on viticulture [7,8]. The increase of the winegrower’s awareness of the need to adapt vineyard management to new climatic conditions has been reported to cope with this issue. Re-assortment of genetic resources plays an important role in this field [9,10,11,12]. Understanding climate risk and improving preparedness to climate impacts—in addition to the development of new technical strategies for vineyard management—is considered at the base of the adaptive capacity of grapevines, and is an intrinsic property supporting resilience of local agricultural systems [13,14,15].

Assuming that limiting factors for high-quality productions, such as air temperature and water availability, vary significantly at the farm’s scale, the grapevine physiological response to microclimate is influenced by the vineyard’s characteristics (e.g., soil, exposure, planting density) and/or management practices (e.g., soil management, pruning, water management). In particular, understanding climate changes at the farm scale is pivotal to optimize efforts for adapting vineyard management practices to climate variability [16]. Consequently, the adoption of agronomical adaptive practices has to be highly vineyard-specific, other than genotype-specific. In this regard, a climate-smart agriculture based on adaptive agricultural practices [17] has to become a priority for the improvement of food quality and security, and for providing mitigation benefits [18,19], while assuring resilience of agro-ecosystems [20,21,22,23,24].

In the Mediterranean basin, one of the largest grapevine production areas in the world, warming has been causing shifts in grapevine phenology, changes in disease and pest patterns, therefore affecting the need of agrochemical use, ripening times and wine styles [1,10], jeopardizing the economic and environmental sustainability for typical and territorial oenological productions [25]. Italy is a Mediterranean country with typical vine productions and important grape-wine growing areas—mainly based on traditional agronomic practices. Nonetheless, vineyard cropping surface areas decreased continuously in the last decades as a result of land-use changes, soil consumption, and agriculture abandonment in marginal areas [20,26,27] or in areas characterized by high anthropogenic pressure [28,29], but also owing to the non-competitive grape and wine quality.

The present study provides an integrated approach grounded on the measure of a set of physical and biological data able to assess the genotype-related response to microclimate variation, with the final aim to orient technical decisions for improving vineyard management. In particular, the specific objectives of the study were: (i) to provide a climate variability assessment in a traditional grape-wine growing study area (Umbria, central Italy) at both large scale and farm scale; (ii) to measure the adaptive response to climate change of seven grapevine varieties (two autochthonous and five international) as far as ripening phenology and berry quality are concerned; and (iii) to measure the effect of changing climate on the main grapevine diseases’ frequency. Our results underline the genotype-specific response of grapevine to climate change, and the usefulness of permanently monitoring micro-climate regimes with the aim to develop specific adaptive viticultural practices for resilient and sustainable productions on a farm’s scale.

## 2. Results 

### 2.1. Multi-Scale Climate Characterization

**Figure 1:**
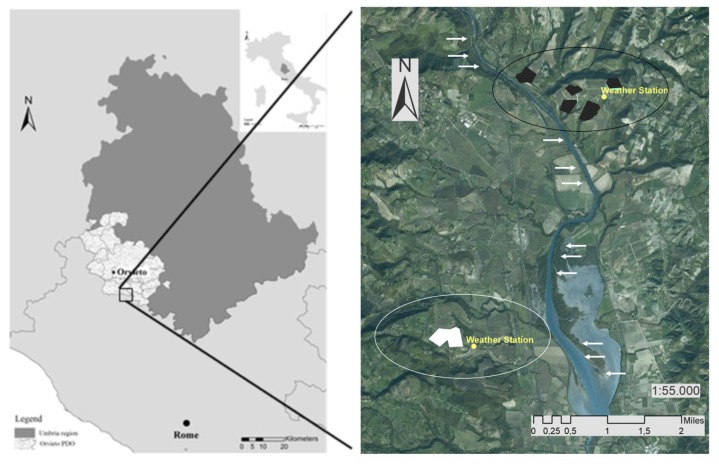
Umbria region (central Italy) and the study grape-wine growing area, the ‘Orvieto’ Protected Designation of Origin (PDO) (left). Localization of the two wineries and tested vineyards (right). White polygons, vineyards of white varieties (Grechetto di Orvieto and Chardonnay), winery of the western sub-area; black polygons, vineyards of red varieties (Aleatico, Merlot, Cabernet sauvignon and C. franc, Sangiovese), winery of the eastern sub-area; yellow circles, position of the on-farm weather stations in each winery; white ellipse, position of the western sub-areas of the PDO; and black ellipse, position of the eastern sub-area of the PDO. Arrows, Tiber River.

The study was carried out in a traditional grape-wine growing area, the Protected Designation of Origins (PDO) ‘Orvieto’ in Umbria region, central Italy (Figure 1), characterized by variable physiographic characteristics (see Section 4.1). Climate characterization interested the whole area, and more specifically, two farms that hosted test vineyards for the assessment of bio-physical indicators.

**Figure 2:**
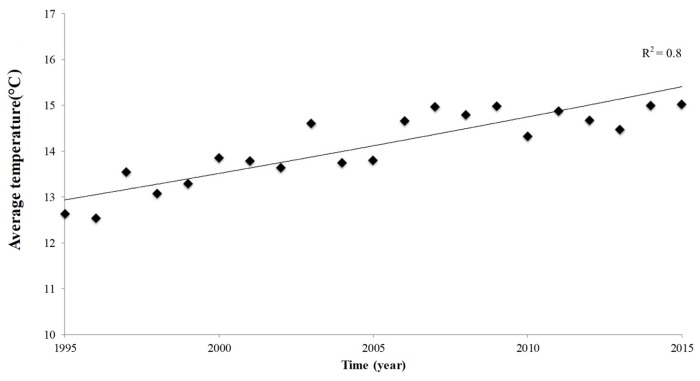
Annual average temperature (°C) in the grape-wine growing area PDO ‘Orvieto’ (1995–2015).

**Figure 3:**
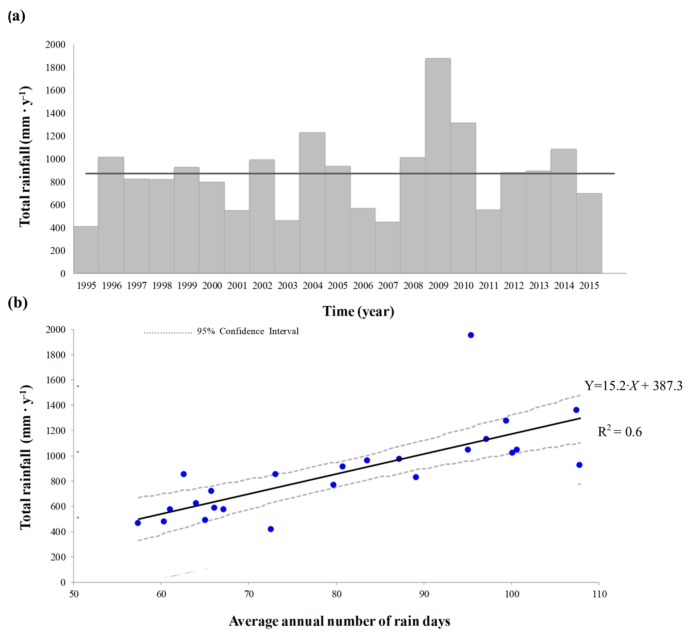
Precipitation in the grape-wine growing area PDO ‘Orvieto’. (**a**) Total precipitation per year and the climatic average (continuous line); and (**b**) linear relationship between total precipitation per year and annual number of rainy days (rain > 1mm/day). Data refer to the period between 1995 and 2015.

The annual mean temperature over the past two decades (1995–2015) increased over time in the study area (Figure 2): during the first decade (1995–2004), the average temperature was 13.5 °C, while in the second decade (2005–2015) it increased up to 14.7 °C. At the same time, an increase in the amount of precipitation was recorded (Figure 3a). The increase in total precipitation correlated (r^2^ = 0.65) with the average number of rainy days (precipitation > 1mm) per year (Figure 3b).

**Figure 4:**
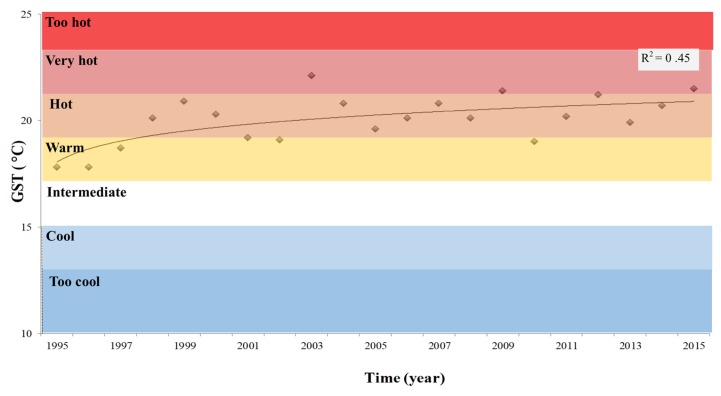
Growing Season average Temperature (GST) (°C) trend in the white variety growing area (western sub-area). Climate class limits according to Nesbitt et al. [30].

In the area where the white grapevine varieties (western sub-area) were cultivated, the Growing Season average Temperature (GST) was 19.7 °C in the first sub-period (1995–2004), and 20.5 °C in the second sub-period (2005–2015). Consequently, classification of local climate regimes in the western area changed from ‘warm–hot’ to ‘hot–very hot’, following a classification provided by Nesbitt et al. [30], and reported in Figure 4. For the red varieties in the eastern sub-area, the GST was instead 19.6 °C during the 2004–2015 period, and the local climate was classified as ‘hot’ (data not shown).

**Figure 5:**
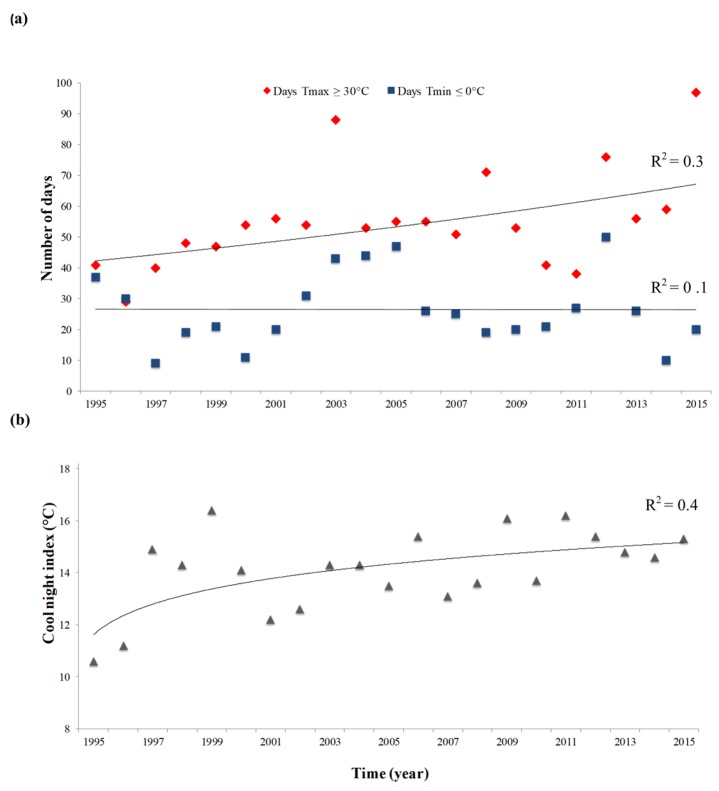
(**a**) Number of days with daily maximum air temperature (Tmax) equal or exceeded to 30 °C (black line and indicators) and with daily minimum temperature (Tmin) equal or below to 0 °C (grey line and indicators) in the white variety growing area (western sub-area). (**b**) Cool night index (CI) trend in the white variety growing area (western sub-area).

Climate analysis showed a trend toward rising temperatures during the summer, as demonstrated by the number of days with daily maximum air temperature (Tmax) exceeding 30 °C (Figure 5a), together with a linear decrease in the number of days with daily minimum temperature (Tmin) equal or below to 0 °C. No significant trends were observed for these two variables in the area of red grape production, situated in the western sub-area (data not shown). The Cool night Index (CI) outlined an increasing trend in the average Tmin in early autumn (Figure 5b). The values of the other bioclimatic indices for the two sub-areas are reported in Table S1a,b. Both Winkler index (WI) and Huglin index (HI) in grapevine growing areas showed increasing values over time. The ombrothermic diagrams (Figure S1) indicate a restricted period of dryness encompassing June to August. Precipitations were concentrated in the growing season (Table S4), while 35–40% of total annual precipitation in both sub-areas occurred out of season. Changes over time in the value of the Seleaninov hydrothermic coefficient are also reported in Table S4.

### 2.2. Ripening Phenology and Berry Quality

**Figure 6:**
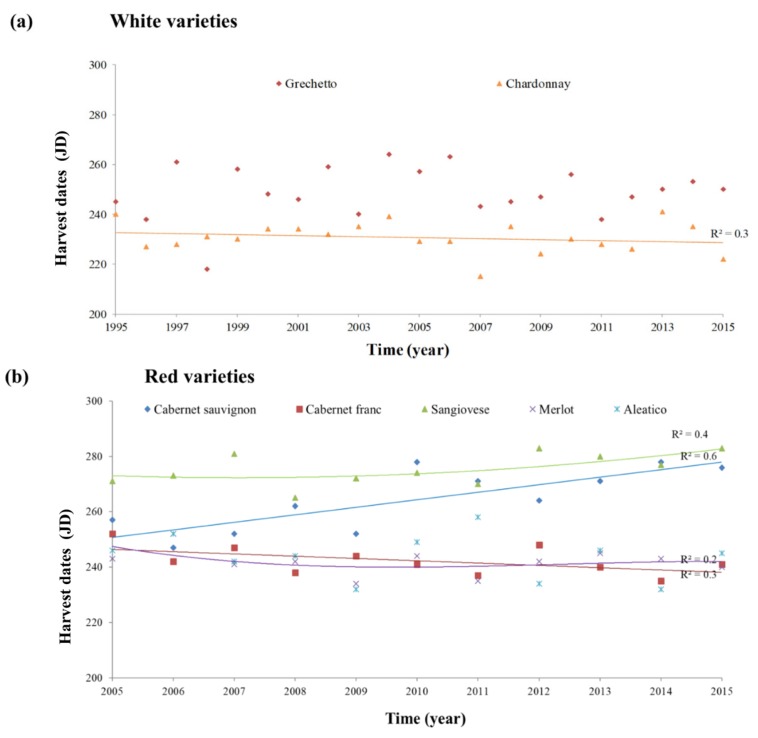
Ripening dates (technological ripening, harvest) and trend lines for the tested grape varieties. (**a**) White grape varieties (western sub-area); and (**b**) red grape varieties (eastern sub-area). Harvest date is reported as Julian days (JD).

The white grape varieties, the international Chardonnay (CH) variety and the autochthonous Grechetto (GR) variety, showed different ripening trends (Figure 6a). In particular, a linear trend in the number of days needed to reach ripening was exhibited by the CH. No trend in harvest dates was recorded for the autochthonous GR, which showed instead the highest variability (JDmax = 264, JDmin = 218) in harvest date. The red grape varieties displayed a different trend (Figure 6b). In particular, the autochthonous Aleatico (A) variety showed no significant trend in the date of ripening (JDmax = 258, JDmin = 232); the Sangiovese and C. sauvignon varieties displayed a delay in fruit maturity as proved by the higher number of days necessary for the technological ripening, whereas Merlot and C. franc showed anticipated harvest dates. Yields over time, provided by winery registers only for a limited set of varieties (CH, GR, and M), are reported in Figure S3, and showed decreasing trends.

**Figure 7:**
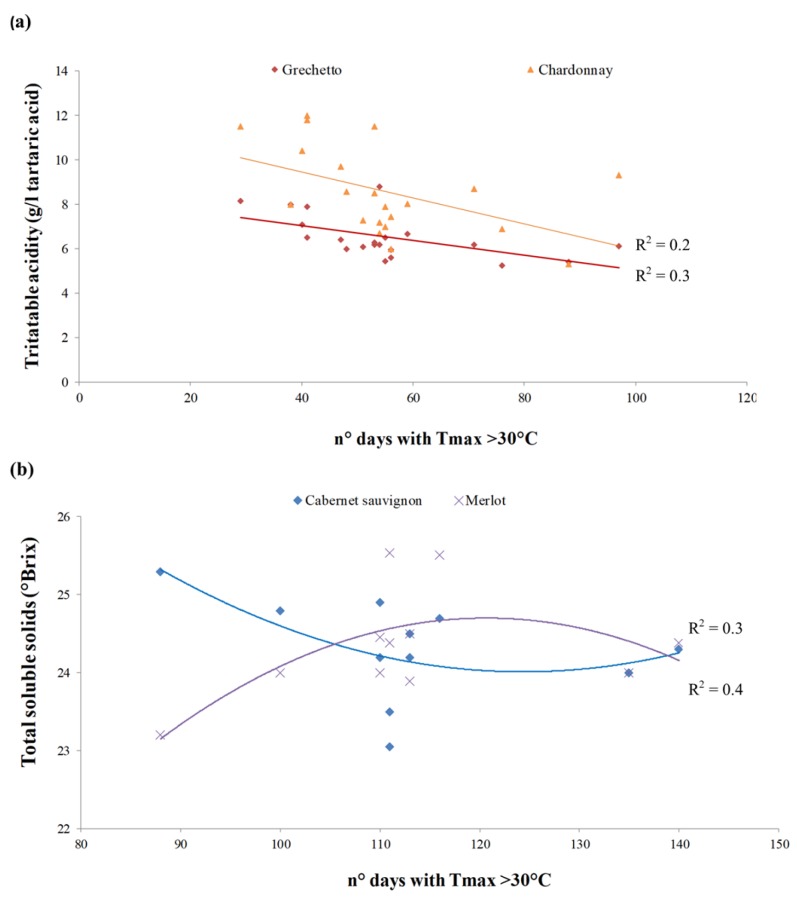
Berry quality and extreme temperature events; (**a**) titratable acidity (g∙L^−1^ tartaric acid) for the white varieties affected by the number of days with maximum temperature (Tmax) equal or exceeded to 30 °C during the growing season; and (**b**) total soluble solids (°Brix) for the varieties Cabernet sauvignon and Merlot related to the number of days with maximum temperature (Tmax) equal or exceeded to 30 °C during the growing season.

In terms of berry quality over the considered time span, the final berry’s total soluble solids (TSS) and titratable acidity (TA) at full ripening in the white varieties were 22.3 ± 1.1 °Brix and 6.5 ± 1.0 mg·L^−1^ tartaric acid for the autochthonous GR variety, and 20.0 ± 1.9 °Brix and 8.6 ± 2.0 mg·L^−1^ tartaric acid, for the CH variety (Table S2a). TA and pH of berry juice at harvest were influenced by thermal regime, expressed by the WI, HI (data not shown), and by the number of days with Tmax > 30 °C, showing a decreasing trend (Figure 7a). 

In the red varieties the amount of TSS in the C. sauvignon (CS) and Sangiovese (S) berries decreased linearly, whereas in C. franc (CF) and Merlot (M) varieties it increased more evidently (Figure S2); for the autochthonous Aleatico (A) variety, no significant trend was identified. Berry TSS final concentration was also evaluated with respect to the number of days with a maximum temperature (Tmax) equal or exceeding 30 °C during the growing season (Figure 7b). Opposite trends in TSS final concentration were exhibited by the red varieties in relation to extreme climate conditions, so that no correlation between these two variables was assessable. Harvest dates and berry qualitative traits were reported for each growing season in the considered time span (Table S2a,b).

**Figure 8:**
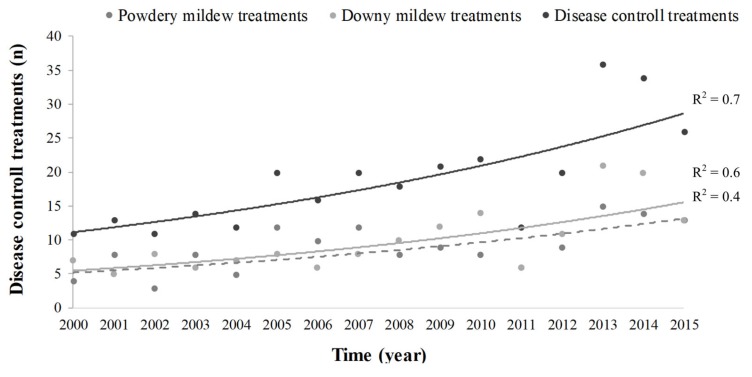
Total disease control treatments (continued black trend lines) and specific treatments for powdery (*Erysiphe necator*) (dotted gray trend lines) and downy mildew (*Plasmopara viticola*) (continued grey trend lines) carried out in each growing season in the western sub-area.

The number of grapevine disease control treatments was analyzed for the white grape growing area. As shown in Figure 8, total phytosanitary treatments, represented by the sum of the powdery and downy mildew treatments, exhibited exponential growth during the last 15 years, with significant differences observed during the growing season (Table S3).

### 2.3. Statistical Analysis

**Figure 9:**
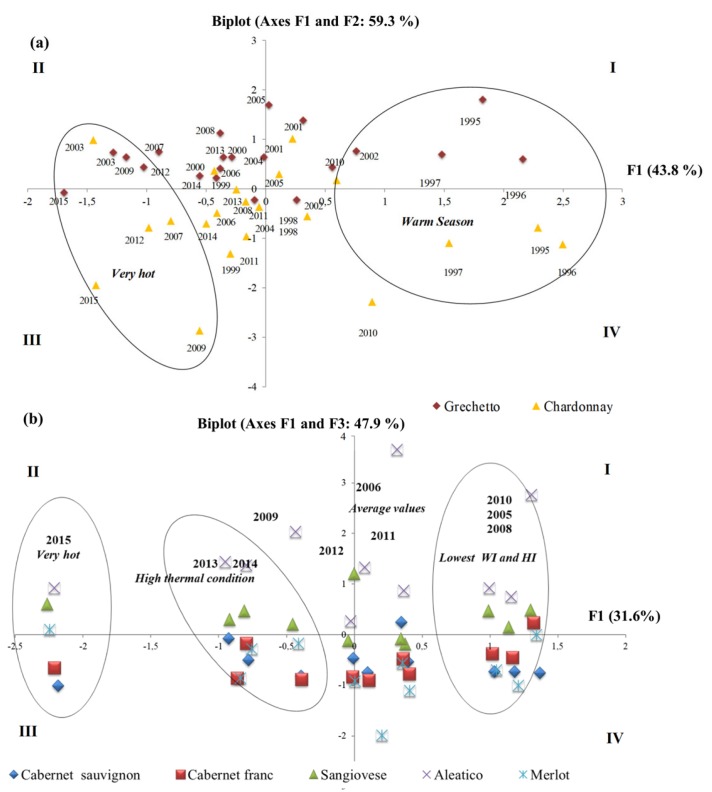
Biplots of the climate indices and berry quality traits along principal components 1 (F1) and principal components 2 (F2) for the white grape varieties (CH and GR) (**a**); and component F1 and F3 for red grape varieties (CS, CF, M, S and A) (**b**). CH, Chardonnay; GR, Grechetto; CS, Cabernet sauvignon; CF, C. franc; M, Merlot; S, Sangiovese; and A, Aleatico. Roman numerals represent the four quadrants of the Cartesian coordinate system.

Pearson’s correlation analysis was carried out for all tested variables: climate data indices, ripening phenology, and grape quality traits, separately for the two grape growing areas. The results revealed that for the white berry varieties (western sub-area) berry quality traits, such as TA and pH of berry juice at harvest, were affected by thermal availability (WI, HI) and extreme summer events, while TSS were significantly correlated with harvest date (Table S2a). In the red grape varieties, on the other hand, a significant correlation between TA and TSS was observed, not with weather indices (Table S2b). A multivariate statistical analysis (PCA) was carried out on white and red grape varieties separately. The PCA extracted two components with 59% of the cumulated variance for white grape varieties and three components for red varieties (66%) (Figure 9a,b, respectively). For the white grapes, principal component 1 (F1) was related to weather variables and bioclimatic indices according to a gradient based on thermal potential (HI, WI, extreme thermal events: negative loadings). Principal component 2 (F2) was related to berry quality according to a gradient based on TSS (positive loading) and TA (negative loading).

Figure 9a,b depicts the PCA result for white and red grape varieties, respectively, with the individual variables projected in the PC1–PC2/PC3 plane. Within the white varieties (Figure 9a), the cv GR predominates in the positive values of F2, while cv CH in the negative ones. These varieties showed changes based on F1 gradient. Exceptionally-dry climatic conditions determine a higher strew across the F2 for these varieties, especially for very hot and warm seasons. For the red grape varieties principal component 1 (F1) was related to the thermal potential of sites (HI, WI, and GST); component 2 (F2) illustrated thermal extreme events (T_max_, N°days with T_max_ ≥ 30, CI, LGS, Total precipitation); on the other hand, component 3 (F3) described berry quality. Scatter plots of the F1–F3 plane of red varieties (Figure 9b), and F2–F3 (data not shown) outlined a comparable climatic gradient. The varieties M, CS, and CF were located on negative values of F3 (III and IV quadrant), whereas the varieties A and S were mainly associated with the I and II quadrants.

## 3. Discussion

The empirical results of this study give an indication of the multiple relationship among grapevine phenology, berry quality (including potential bunch healthiness), and climate change in a traditional region devoted to high quality wine productions in central Italy. An integrated evaluation of physical (climate factors) and biological variables (berry ripeness date, TSS and TA content) is proposed in this study with the aim to inform management practices ranging from choice of grapevine varieties to planning water resource management, phytosanitary defense, and harvest organization. It is widely recognized that analysis of time-series climate data is a key approach for the adoption of adaptation strategies in climate sensitive agro-systems, including vineyards [10,31,32,33]. This approach allows continuous environmental monitoring and design of future climate scenarios [12,16] in wine-grape growing areas, identifying the suitability of grapevine varieties to local conditions, and climate impact on grapevine production [1,8]. Results of this study also recognize the importance of a local-scale assessment of climate variability [34,35] shedding light on the main effects of climate change. 

Air temperature was recognized as a key climate variable regulating the environment–genotype relationship in *Vitis vinifera* [2,30,36,37], being that the growth habit of this species highly susceptible to thermal regime. Moreover, temperature variability may become the prevalent factor affecting wine’s quality.

Overall, long-term climate observations in a traditional grape-wine growing area highlighted important weather modifications over time. In particular, warming, variations in precipitation patterns, and a progressive increase in extreme summer events were the main changes observed during 1995–2015. The study area, classified as ‘hot-warm’ climate in 1995–2015, according to GST values [30], exhibited an increasingly linear trend in the GST (1.4 °C in the last 15 years). Estimated warming of the grapevine vegetative period is in accordance with what was observed in other traditional wine growing areas [1,2,38], or predicted by climate models [7,12]. The WI and HI indices, that provide information about temperatures necessary for vine growth, grape ripening, and grape oenological potential [39], also increased over time like in other Mediterranean areas [40,41]. This process may lead to a local rearrangement of crop varieties. Contrary to what was observed elsewhere [15,42], the increase in temperature was not coupled with an extension of the drought period, usually leading to increased irrigation needs in the vineyard [43], and consequently to the necessity to adjust vineyard management practices for semi-arid regions. Indeed, an increase in total precipitation and numbers of rainy days (rain > 1mm) was recorded in this grape-wine growing area, in line with earlier results provided by Ramos et al. [40]. More specifically, precipitation frequency and amount did not show any significant trend against time as observed in Jones et al. [1] and Tomasi et al. [41]. This evidence was proved by analysis of the HTC calculated for the two sub-areas. The occurrence of a high amount of precipitation out of season (hydrological winter), given the high soil organic matter typical of both sub-areas [44], can indicate the replenishment of soil water content. A relatively high amount of soil organic matter, together with the values of HTC prove the effectiveness of precipitation in the growing season, preventing the need of vineyard systematic irrigation, and with a consistent increase of pest and diseases risk. Given this fact, while a more sustainable use of water resources may indicate the occurrence of sustainability condition in the agro-ecosystem, the increase of agrochemical need endangers this objective. Therefore, ombrothermic graphs are a key tool to profile micro-climate regimes in the vineyard and to plan adaptation strategies at soil or canopy level [30].

A special focus is devoted to analyzing the occurrence of extreme climate events in grape-wine growing areas (e.g., late spring frost, extended hot days, extreme rainfalls) [2,14,37,40]. In this regard, we measured the increase over time in the frequency of extreme summer events. Growing season extreme maximum temperature (number of days with T_max_ > 30 °C) during last phases of berry ripening increased significantly over the past 20 years. The warmest growing seasons were 2003 and 2015. In particular, the 2003 vintage was classified as ‘too hot ‘ and ‘moderately dry’, according to the classification by Tomasi et al. [41]. Furthermore, the 2015 vintage was ‘very hot’ but with most of the seasonal precipitation centered in the ripening phonological phase from July to September. Physiological implications of the simultaneous occurrence of high temperatures and excessive water availability in the late phases of berry growth are known and well documented [45,46,47] and usually result in irreversible berry damages, such as berry crack and rot [48]. Some vine responses may be mediated by the indirect effect of atmosphere on the soil system, by modifying its physical traits, but also its microbial composition. This relation should be investigated in further research. 

One of the most frequently underestimated consequences of climate variability is the modification of variety phenology, that should be a rather stable genetically-determined character in the same growing environment [49]. Grapevine phenology has been monitored in many grape-wine growing regions on reference varieties compared with temperature increases [1,9,50]. Recently, grapevine phenology has been modeled to identify significant correlations with changes in Mediterranean climate regimes [51]. Some authors have highlighted how grapevine varieties’ harvest date has none to moderate relationship to climate trends [40], or even major disagreement with climate variable; this phenological phase depends on vineyard management [51]. 

Our investigation proved the influence of climate variability on ripening phenology and consequently harvest date, and also on berry ripening indices, like TSS and TA. The response was highly genotype-specific, differing among international and native grapevine varieties and between white grape and red grape varieties. In the last years, there is an increasing interest by winegrowers in adopting autochthones genotypes, being landraces known to optimize genotype–environment interaction [52,53] and to exhibits greater phenotypic plasticity to climate conditions [54,55]. Local genetic resources have a role in sustainable viticulture, allowing rich high quality grape and oenological products with minimal outside-farm input requirements [53]. Concerning the ripening phase, the two tested autochthonous varieties, the white grape cv Grechetto and the red grape cv Aleatico, showed annual fluctuations in the harvest date, irrespective of warming. In general, the international varieties exhibited adaptive traits, resulting in a strict correlation of ripening phenology (occurrence of technological ripening) with climate change, although with a different pattern among the genotypes. In fact, genotype response was reflected in both advanced and delayed harvest dates. For example, a different behavior was exhibited within the tested red grape varieties, all classified as mid to late season varieties [56]. Grapevine varieties clustered into climate-maturity groupings, according to Jones [2]. In particular, cv C. Sauvignon and cv Sangiovese, classified as suitable for ‘warm climate maturity’ ranges (GST, 17–19°C), while cv C. franc and cv Merlot classified for ‘intermediate’ to ‘warm climate maturity’ ranges (GST, 15–17 °C and 17–19 °C, respectively). Our study proved that climate maturity grouping may change over time. This genotype-specific response in ripening phenology implies that the precision management of harvest has to deal with the genotype different response to climate change.

Berry biochemistry was correlated to thermal regime variation. This result has to be discussed separately for the white and red varieties, under different oenological targets. The PCA results helped to understand which climate variables mostly affect berry ripening at farm level and within variety groups. The tested white varieties exhibited linear reduction in berry TA in respect to the increasing number of extremely hot days (Tmax > 30 °C) in the ripening period, below the optimum for white vinification [57], as found by other authors [2]. All the tested red grape varieties showed different trends for sugar accumulation (TSS) and total acidity (TA). In particular, the varieties Sangiovese and C. sauvignon, belonging to the first cluster (‘warm climate-maturity’; optimum GST, 17–19 °C), exhibited a clear trend to postponement of the harvest date and reduction of TSS. In the second cluster group, the cv C. franc and cv Merlot, indeed, anticipate harvest time and increase TSS accumulation. Nonetheless, TSS concentration—a key biochemical parameter of red vinification [57]—did not exhibit certain trend (increase vs decrease) with respect to extreme temperature frequency, proving the importance of specific investigation to assess carbohydrate allocation under climate variability. The measured high variability may be explained with the strong genotype-related response to climate change, having berry quality a multiple and complex nature controlled by many interconnected genetic factors [58], that need to be investigated further.

Climatic alteration also affects the bunch potential healthiness. Climate variability affects the patterns of plant diseases by changing the geographical distribution of pathogens or affecting the physiology of the host–pathogen interaction or the pathogen evolution under altered climatic conditions [59,60,61]. The potential impact of climate change on the use of agrochemicals has both environmental and economic consequences. According to Salinari et al. [62], predictable models show an increase in disease pressure from *P. viticola*; on the other hand, they show a significant decrease of powdery mildew epidemics in the last century [57]. Several disease models have been used to simulate future scenarios of plant pathogens epidemics on grape with climate changes [59,62]. In the study area, the total number of phytosanitary treatments increased exponentially in the last 15 years, particularly against the downy mildew. This evidence suggests the importance of the on-farm monitoring and the opportunity to develop decision support innovation for precision viticulture and sustainable productions.

## 4. Materials and Methods 

### 4.1. Study Area

The study was carried out in a traditional grape-wine growing area in central Italy, the Protected Designation of Origins (PDO) ‘Orvieto’ in Umbria region, central Italy (Figure 1, left). The study area is part of a temperate region with lower thermo-Mediterranean thermotype and low humid ombrotype [63]. Climate regime is typically Mediterranean Csa subtype (Temperate, Dry summer, Hot summer) with precipitations (ranging from 950 to 1200 mm yr^−1^) concentrated in autumn and spring, restricted summer dryness period and mild air temperatures [64]. The selected study area includes 18 municipalities and extends 736 km^2^ alongside the Tiber River, that split the area in two homogeneous sub-areas (western and eastern side of the Tiber River) with variable physiographic characteristics. In particular, Western and Eastern areas of the Tiber River differ in elevation (100–200 m a.s.l. and 220–380 m a.s.l., respectively) and soil typology (volcanic vs sedimentary) [65]. One representative winery for farm size, Utilized Agricultural Area (UAA) and management model (conventional viticulture with integrated pest management) was selected in each sub-area (Figure 1, right). Representative and homogeneous vineyards were identified within each farm. The selected vineyards were also homogeneous for age (year of planting: 1995), training system (vertical shoot positioning training system, either cordon or Guyot), and employed rootstocks (mainly Paulsen 1103). All the selected viticultural surface areas had a southern exposition, and were planted on weakly mineral and moderate deep soil (regosol) [66] characterized by limestone gravels in a sandy matrix, rounded clasts, mainly cemented, containing low to moderate water reserve. This soil typology is appropriate for grapevine cultivation and high-quality productions [67].

### 4.2. Experimental Design, Conceptual Framework and Statistics

The present study considered 7 grapevine varieties, both international (5 varieties), Merlot (M), Cabernet Sauvignon (CS), Cabernet Franc (CF), Chardonnay (CH), Sangiovese (S) and autochthonous (2 varieties), Aleatico (A) [67], and the native Grechetto di Orvieto (GR) [52]. These varieties are representative of the amphelographic base of the central Italy oenological products. The white varieties (CH and GR) were predominant in the western sub-area; red varieties (M, CS, CF, S, and GR) were concentrated in the eastern one.

Considering two sub-areas was appropriate when testing the behavior of both white and red varieties, that are respectively predominant in the western and eastern side of the Tiber River (Figure 1, right). Climate characteristics were investigated on a large but spatially-detailed scale, offering a separate analysis for the whole PDO ‘Orvieto’ district and for the two study sub-areas. Climate analysis at the large scale was carried out based on time-series (1995–2015) data of average temperature and precipitation provided by public regional weather stations installed in the PDO. At a detailed scale, climate data of temperature and precipitation were provided by on-farm weather stations. In particular, climatic variables for the whole PDO came from a weather station (Code: 06SENSI) based in the center of the area of production and belonging to a regionally controlled network of stations that provide open-source data (http://www.parco3a.org/am/rilevazioni.aspx). The following climate variables were recorded: daily average temperature, maximum and minimum temperature, and precipitations. Daily data were organized on a month or year scale. Precipitations were split in two data sets depending on the hydrological periods: the hydrological summer (growing season; May-October northern hemisphere) and hydrological winter (off-season; November–April northern hemisphere) as reported in OIV, 2015 [34]. Data were available on line for the considered time span (1995–2015). At farm level, two whether stations (Netsens s.r.l Italy, model MeteoSense 2.0) provided the same daily climatic variables, periodically downloaded by the winegrowers. Single measurements were kindly provided by each winery for the periods 1995–2015 and 2004–2015, respectively, in the western and eastern sub-areas. To define climate change effects on grapevine varieties’ behavior and performances, historical data provided by each winery for variety phenology and grape quality traits (expressed as sugar and organic acid content), were also acquired. Statistical analysis was run on empirical data with the aim to describe the effect of climate changes on grapevine phenology and grape quality.

A Pearson linear correlation analysis was performed for all the variables included in this study: climate data indices, historical data of phenology, and grape quality, distinctively for the two grape-growing areas. The results indicate pair-wise significant correlations testing at *p* < 0.05.

A Principal Component Analysis (PCA) was run with the aim to reduce the number of variables for data modeling, in particular to reduce multicollinearity existing among the bioclimatic indices (see Table 1), and to understand how and which climatic parameters affect grape quality, providing an intrinsic (re)classification of harvest time. Statistical analysis was developed using R package—release 3.5.2 (http://www.R-project.org/).

### 4.3. Bioclimatic Indices and Agro-Phenological Trait Assessment

Air temperature data recorded continuously over the last 20 years (1995–2015) was processed to calculate bioclimatic indices, based on daily temperature profile (minimum, maximum, and daily average) characterizing land suitability to grapevine growth and wine production in a given geographical area. Bioclimatic indices adopted in this study (Table 1) are suited to classify climate regimes in any given grape-growing area according to the Multicriteria Climatic Classification System (MCC) [68].

In addition, the average month precipitation and monthly average temperature were used to elaborate ombrothermic diagrams covering a period of 20 years [1].

Agro-phenological data were available for the last 20 years (1995–2015) for the white grapevine varieties (vineyards located in the western sub-area), and for the last 10 years (2004–2015) for the red grapevine varieties (vineyards located in the eastern sub-area). In both cases, given the vineyard age, the investigated time span represents the period of vine’s full maturity. Data on variety phenology—in our study the full ripening stage following BBCH classification (technological ripening) [75]—and qualitative metrics for berry quality, like total soluble solids (TSS) (°Brix), titratable acidity (TA) (mg Tartaric acid l-1), and pH, were acquired by winery registers. Owners of both wineries have registered the occurrence of the harvest date for each variety over time. Furthermore, variables related to grape composition were also determined for each variety at harvest, by standard on-farm determination (refractometer and winery’s laboratory analysis). Wineries also provided historical series of yield records for some varieties, only (CH, GR, and M). Data were analyzed for the white and red grapevine varieties separately. Data on plant protection, were derived from the number of total pesticide treatments applied yearly (2000–2015) against Downy mildew (*Plasmopara viticola*) and Powdery mildew (*Uncinula necator*), were acquired. Historical records on the number of treatments were also provided by wineries.

## 5. Conclusions

This overall analysis allows qualifying the thermal responses of some local and international grapevine varieties to climate change on a local scale. The phenology of grapevine and berry ripening indices are extremely sensitive to climate and highly genotype-specific. Climate characterization and permanent monitoring of phenological traits and berry biochemistry in accordance to international protocols, are efficient tools to define environmental vulnerability (or resilience) of worldwide known grape-wine growing areas. Integrated data analysis allows identification of trends and correlations between grapevine phenology and climate variability, addressing strategies for future planning of viticulture practices, in line with local climate variability. Local-scale knowledge of climate impact on grapevine phenology should be integrated into regional models predicting susceptibility of vines to pest diseases, improving goodness of fit of local-scale models grounded on information collected with dedicated on-farm surveys. Innovation technologies may also increase on-farm availability of integrated climate information, and improving winegrowers’ environmental awareness. In other words, local climate should be increasingly seen as a key factor of farms’ competitiveness. Based on local knowledge, in the scenario of predicted climate change, winegrowers could better address the adjustment of variety assortment that assures the maintenance of grape quality attributes, contributing to high quality wines and market competiveness. Finally, the comprehensive analysis of on-farm micro-climatic traits may contribute significantly to identification of both ecological and economic niches where viticulture could be successful regardless global adverse climate conditions.

## Figures and Tables

**Table 1:** Temperature-based bioclimatic indices used for climate characterization in the studied grape-wine growing areas.

Climate index	Abbreviation	Formula	Unit	Source
Length of the growing season	LGS	Σ n day Tm > 10°C	GDD	[69,70]
Growing season average temperature	GST	Tm (1st April–31th October, in the northern hemisphere).	°C	[69,70]
Winkler index	WI	$\mathrm{WI}\;=\;\overset{31\;Oct\;}{\underset{1\;Apr}{\sum }}\left(Tm\;-\;10\;{}^{\circ}C\right)$ where Tm is the daily average temperature.	GDD	[71]
Huglin index	HI	$HI\;=\;\overset{30Sep}{\underset{1\;Apr}{\sum }}\;\frac{\left[\;\left(Tm\;-\;10\right)+\left(Tmax\;-10\right)\right]}{2}\cdot d$ where Tm is the average air temperature; Tmax maximum air temperature; d length of day coefficient. d = 1.03 in the study area.	GDD	[39,68]
Cool night index	CI	Tmin in the month of September (northern hemisphere) (average of minima)	°C	[68]
Frost days	-	days Tmin ≤ 0 °C	n.	[40,72]
Ice days	-	days Tmax ≤ 0 °C	n.	[40,72]
Moderate hot days	-	days Tmax ≥ 25 °C	n.	[2]
Maximum temperature prior to harvest		Tmax month before harvest	°C	[68]
Extreme warm events	-	days Tmax ≥ 30 °C	n.	[2,72,73]
Seleaninov hydrothermic coefficient	HTC	$\mathrm{HTC}\;=\;\overset{30Sep}{\underset{1\;Apr}{\sum }}\;P/\mathrm{WI})\;\times \;10$ where P is Total precipitation in the growing season; WI, Winkler index		[74]

## Supplementary Materials

The following are available online at https://www.mdpi.com/2223-7747/8/5/121/s1, Figure S1: Ombrothermic diagrams of the western sub-area (**a**) and of the eastern sub-area (**b**); Figure S2: Total soluble solids (°Brix) trends for red varieties related to seasons; Figure S3: Trend in grapevine yields over the period 2004–2015 for some white and red varieties; Table S1: Climate data and bioclimatic indices for the western (**a**) and eastern (**b**) grape growing area of PDO ‘Orvieto’ (central of Italy); Table S2: Grapevine harvest dates and berry qualitative parameters. (**a**) White varieties and (**b**) red varieties; Tables S3: Pearson’s correlation matrices for white variety growing area (western sub-area) (**a**) and red variety growing area (eastern sub-area) (**b**), and significance between Pearson’s coefficient correlation (ns, not significant; **p* < 0.05; ***p* < 0.01); Table S4: Precipitation in the hydrological periods and Seleaninov hydrothermic coefficient (HTC).
