# Supplementary material for: Assessing Impacts of Climate Change on Phenology and Quality Traits of Vitis vinifera L.: The Contribution of Local Knowledge
Source: Plants (Basel). 2019 May 9;8(5):121. doi: 10.3390/plants8050121 (PMC6571897; doi:10.3390/plants8050121)
Supplement: Supplementary file 1 [file plants-08-00121-s001.pdf]

Supplementary material

Table S1. Climate data and bioclimatic indices for the western (**a**) and eastern (**b**) grape growing area of PDO 'Orvieto' (central of Italy). Indexes calculated for the period 1995–2015 and 2004–20015, for the western and eastern sub-areas, respectively. Western sub-area: white grape varieties. Eastern sub-areas: red grape varieties. LSG, length of the growing season; GST, growing season average temperature, Tmin, yearly mean Tmin; Tmax, yearly mean Tmax, WI, Winkler index, HI, Huglin index; CI, cool night index.

(a)

Year	LGS	GST	GST Class limits (Nesbitt et al.) [45]	Tmin	Tmax	WI	HI	HI class limits (Tonietto and Carbonneau) [36]	CI	CI class limits (Tonietto and Carbonneau) [36]	Tmax (month prior harvest date)	N°days Tmax ≥ 30 °C	N°days Tmax ≥ 25 °C	N° days Tmin ≤ 0 °C	N° days Tmax ≤ 0 °C
1995	197	17.8	warm	7.9	18.3	1795	2041	temperate	10.6	very cool nights	22.9	41	79	37	1
1996	204	17.8	warm	8.3	18.8	1510	1966	temperate	11.2	very cool nights	24.4	29	79	30	0
1997	190	18.7	warm	9.1	19.2	1760	2176	warm temperate	14.9	temperate nights	28.6	40	113	9	0
1998	209	20.1	hot	10.1	19.1	2294	2524	warm	14.3	temperate nights	27.4	48	97	19	0
1999	211	20.9	hot	10.7	19.4	2485	2645	warm	16.4	temperate nights	28.8	47	121	21	1
2000	213	20.3	hot	10.1	20.3	2456	2700	very warm	14.1	temperate nights	30.8	54	121	11	0
2001	202	19.2	hot	9.5	19.8	2366	2497	warm	12.2	cool nights	28.1	56	103	20	0
2002	204	19.1	hot	9.6	20.5	1818	2319	warm temperate	12.6	cool nights	25.7	54	89	31	1
2003	202	22.1	very hot	9.7	21.1	2720	3028	too Hot	14.3	temperate nights	30.8	88	133	43	0
2004	207	20.8	hot	9.2	20.3	2305	2473.59	warm	14.3	temperate nights	27.5	53	105	44	2
2005	211	19.6	hot	8.7	19.7	2216	2616	warm	13.5	cool nights	27.7	55	109	47	0
2006	214	20.1	hot	9.7	19.2	2502	2683	warm	15.4	temperate nights	28.8	55	115	26	0
2007	213	20.8	hot	10.9	19.6	2597	2848	very warm	13.1	cool nights	29.1	51	127	25	0
2008	212	20.1	hot	9.8	19.1	2200	2437	warm	13.6	cool nights	30.4	71	92	19	0
2009	214	21.4	very hot	10.9	19.5	2535	2741	very warm	16.1	temperate nights	29.8	53	118	20	0
2010	208	19.0	hot	9.3	18.3	2108	2354	warm temperate	13.7	cool nights	28.1	41	100	21	0
2011	203	20.2	hot	10.2	19.9	2250	2475	warm	16.2	temperate nights	30.6	38	115	27	0
2012	208	21.2	very hot	10.0	19.8	2541	2759	very warm	15.4	temperate nights	31.5	76	118	50	5
2013	208	19.9	hot	9.9	18.8	2317	2507	warm	14.8	temperate nights	29.9	56	106	26	0
2014	210	20.7	hot	10.6	20.9	1918	2582	warm	14.6	temperate nights	28.4	59	105	10	0
2015	212	21.5	very hot	10.3	24.7	2293	2975	very warm	15.3	temperate nights	27.9	97	153	20	0

(b)

Year	LGS	GST	GST Class limits (Nesbitt et al) [45]	Tmin	Tmax	WI	HI	HI class limits (Tonietto and Carbonneau) [36]	CI	CI class limits (Tonietto and Carbonneau) [36]	Tmax (month prior harvest date)	N°days Tmax ≥ 30 °C	N°days Tmax ≥ 25 °C	N° days Tmin ≤ 0 °C	N° days Tmax ≤ 0 °C
2004	187	20.3	hot	9.9	20.9	1689	2183	warm temperate	14.3	temperate nights	26.8	98	119	78	0
2005	211	19.3	hot	8	20.6	1849.3	2462.6	warm	14.4	temperate nights	28.8	88	130	73	0
2006	214	19.9	hot	8.8	21.5	1910.2	2556.3	warm	14	temperate nights	29.9	113	135	52	0
2007	211	20	hot	9.1	22	1971.8	2638.9	warm	12.5	cool nights	29.8	135	147	25	0
2008	293	18.9	warm	9.3	21.7	1864.9	2475.3	warm	13	cool nights	30.9	110	155	38	0
2009	208	19.8	hot	9.8	21.8	1967.1	2568	warm	15.5	temperate nights	31.9	113	142	7	0
2010	207	18.6	warm	8.7	20.1	1724.4	2303.8	warm temperate	12.4	cool nights	23.4	110	120	30	0
2011	257	19.5	hot	9.1	22.5	1933.4	2586.7	warm	11.8	very cool nights	30.7	116	189	35	0
2012	230	19.6	hot	8.9	21.7	1892.3	2518	warm	16.5	temperate nights	30.9	111	137	28	2
2013	201	19.9	hot	9.7	20.8	1980.7	2699	warm	15.9	temperate nights	29.3	111	131	25	0
2014	212	19.3	hot	10.2	21.3	1994.6	2776	warm	14.7	temperate nights	26.9	100	133	9	0
2015	210	20.5	hot	9.8	21	2252.5	3038	very hot	15	temperate nights	28.6	140	132	12	0

Table S2. Grapevine harvest dates and berry qualitative parameters. (**a**) White varieties; (**b**) red varieties. Data refer to the period 1995–2015 and 2004–2015. for white and red grape varieties, respectively. Data refer to the period 1995–2015 and 2004–2015. for white and red grape varieties, respectively. CH. Chardonnay; GR. Grechetto; CS. Cabernet sauvignon; CF. C. franc; M. Merlot; S. Sangiovese; A. Aleatico. TSS. total soluble solids; TA. titratable acidity.

(a)

	**Harvest dates**		**Yield**		**TSS**		**TA**		**pH**	
	**GR**	**CH**	**GR**	**CH**	**GR**	**CH**	**GR**	**CH**	**GR**	**CH**
**Year**	**(JD)**		**q/vine**		**(°Brix)**		**(g Tartaric Acid· l^{-1})**			
2005	257	229	40	74	24.4	22.2	5.5	7.0	3.3	3.3
2006	263	229	84	74	21.0	21.2	6.5	7.9	3.2	3.3
2007	243	215	80	85	23.2	20.9	6.1	7.3	3.4	3.4
2008	245	235	53	15	23.6	20.9	6.2	8.7	3.5	3.4
2009	247	224	26	92	23.8	16.6	6.3	11.5	3.6	3.2
2010	256	230	58	49	21.7	16.0	7.9	12.0	3.2	3.1
2011	238	228	52	86	23.0	19.8	8.0	8.0	3.2	3.3
2012	247	226	38	48	21.6	19.2	5.3	6.9	3.3	3.4
2013	250	241	46	42	21.8	21.4	6.0	7.5	3.4	3.4
2014	253	235	53	46	22.1	20.6	6.7	8.0	3.3	3.3
2015	250	222	62	46	21.7	17.4	6.1	9.3	3.3	3.2
**Mean**	249.9	228.5	53.7	59.7	22.5	19.7	6.4	8.6	3.3	3.3
**SD**	7.1	7.0	17.2	23.9	1.1	2.1	0.9	1.7	0.1	0.1
**Max**	263.0	241.0	84.0	92.0	24.4	22.2	8.0	12.0	3.6	3.4
**Min**	238.0	215.0	26.0	15.0	21.0	16.0	5.3	6.9	3.2	3.1
**Range**	25.0	26.0	58.0	77.0	3.4	6.2	2.8	5.1	0.4	0.3

(b)

Year	Harvest dates (JD)					TSS (°Brix)					TA (g Tartaric Acid· l^{-1})					pH				
	CS	CF	S	M	A	CS	CF	S	M	A	CS	CF	S	M	A	CS	CF	S	M	A
2005	257	252	271	243	246	25.3	24.0	23.8	23.2	20.5	6.9	6.5	7.5	5.5	7.5	3.5	3.5	3.3	3.6	3.3
2006	247	242	273	252	252	24.2	23.9	23.2	24.5	20.0	7	6.4	7.2	6	7.8	3.5	3.5	3.4	3.6	3.3
2007	252	247	281	241	242	24.0	24.2	23.8	24.0	21.1	7.3	6.2	7.6	6.2	7	3.5	3.5	3.4	3.6	3.4
2008	262	238	265	242	244	24.2	23.7	24.1	24.0	20.8	6.3	6.3	8.6	6.45	7.8	3.6	3.5	3.4	3.6	3.3
2009	252	244	272	234	232	24.5	24.4	24.9	23.9	17.8	6.6	6.4	8.3	7.3	9.5	3.6	3.6	3.3	3.4	3.2
2010	278	241	274	244	249	24.9	23.5	23.8	24.5	18.5	6.5	6.6	7.3	7.2	10.8	3.7	3.3	3.3	3.4	3.1
2011	271	237	270	235	258	24.7	25.4	24.7	25.5	17.0	8.9	7.1	7.8	6.7	13.3	3.5	3.5	3.5	3.4	3.1
2012	264	248	283	242	234	23.1	23.5	21.6	25.5	17.7	6.2	6.1	8.4	6.9	8	3.6	3.6	3.1	4.1	3.3
2013	271	240	280	245	246	23.5	24.7	23.7	24.4	19.2	6.6	6.4	8.1	7.0	7.3	3.4	3.6	3.4	3.7	3.1
2014	278	235	277	243	232	24.8	25.1	24.1	24.0	20.1	6.4	6.5	7.9	6.8	7.9	3.5	3.3	3.3	3.5	3.2
2015	276	241	283	240	245	24.3	24.9	23.8	24.4	20.4	6.2	6.3	8.3	7.1	8.2	3.61	3.4	3.2	3.2	3.3
**Mean**	264.4	242.3	275.4	241.9	243.6	24.3	24.3	23.8	24.4	19.4	6.8	6.4	7.9	6.7	8.6	3.5	3.5	3.3	3.6	3.2
**SD**	11.2	5.1	5.9	4.8	8.3	0.6	0.7	0.9	0.7	1.4	0.8	0.3	0.5	0.6	1.9	0.1	0.1	0.1	0.2	0.1
**Max**	278.0	252.0	283.0	252.0	258.0	25.3	25.4	24.9	25.5	21.1	8.9	7.1	8.6	7.3	13.3	3.7	3.6	3.5	4.1	3.4
**Min**	247.0	235.0	265.0	234.0	232.0	23.1	23.5	21.6	23.2	17.0	6.2	6.1	7.2	5.5	7.0	3.4	3.3	3.1	3.2	3.1
**Range**	31.0	17.0	18.0	18.0	26.0	2.3	2.0	3.3	2.3	4.1	2.7	1.0	1.4	1.8	6.3	0.3	0.3	0.3	0.9	0.3

Tables S3. Pearson's correlation matrices for white variety growing area (western sub-area) (**a**) and red variety growing area (eastern sub-area) (**b**) and significance between Pearson's coefficient correlation (ns. not significant; **p* < 0.05; ***p* < 0.01). TA. titratable acidity; TSS. total soluble solids; CI. cool night index; GST. growing season average temperature; HI. Huglin index; LSG. length of the growing season; Tmin. yearly mean Tmin; Tmax. yearly mean Tmax. WI. Winkler index.

(a)

	TA	Year	TSS	Harvest date	CI	GST	HI	LGS	Days Tmax ≥ 30 °C	Powdery mildew treatments	Downy mildew treatments	Disease control treatments	pH	Total rainfall	Tmax	Tmin	WI
**TA**	1																
**Year**	−0.1	1															
**TSS**	−0.8	−0.1	1														
**Harvest date**	−0.4	0.0	0.4	1													
**CI**	−0.1	0.5	−0.1	0.0	1												
**GST**	−0.4	0.5	0.0	−0.1	0.7	1											
**IH**	−0.4	0.5	0.1	−0.1	0.6	0.9	1										
**LGS**	−0.2	0.5	0.1	−0.1	0.3	0.5	0.6	1									
**Days Tmax ≥ 30°C**	−0.4	0.5	0.1	0.0	0.3	0.7	0.7	0.3	1								
**Powdery mildew treatments**	−0.1	0.7	0.0	−0.1	0.2	0.2	0.4	0.4	0.3	1							
**Downy mildew treatments**	0.2	0.7	−0.2	0.1	0.2	0.0	−0.1	0.2	0.1	0.7	1						
**Disease control treatments**	0.1	0.8	−0.2	0.0	0.2	0.1	0.1	0.3	0.2	0.9	0.9	1					
**pH**	−0.5	0.2	0.4	−0.1	0.3	0.5	0.5	0.3	0.4	0.1	−0.1	0.0	1				
**Total rainfall**	0.4	0.3	−0.2	0.1	0.3	0.1	−0.1	0.5	0.0	0.0	0.4	0.3	−0.1	1			
**Tmax**	−0.2	0.4	0.0	0.0	0.3	0.6	0.6	0.2	0.7	0.1	0.0	0.1	0.2	0.0	1		
**Tmin**	−0.2	0.5	0.0	−0.1	0.7	0.8	0.7	0.6	0.3	0.3	0.3	0.3	0.4	0.2	0.3	1	
**WI**	−0.4	0.3	0.1	−0.1	0.5	0.8	0.9	0.5	0.5	0.1	−0.4	−0.2	0.6	−0.3	0.2	0.6	1

Pairwise two-sided p- values:

	TA	Year	TSS	Harvest date	CI	GST	HI	LGS	Days Tmax ≥ 30 °C	Powdery mildew treatments	Downy mildew treatments	Disease control treatments	pH	Total rainfall	Tmax	Tmin	WI
**TA**																	
**Year**	ns																
**TSS**	**	ns															
**Harvest date**	*	ns	*														
**CI**	ns	**	ns	ns													
**GST**	ns	**	ns	ns	**												
**HI**	**	**	ns	ns	**	**											
**LGS**	ns	**	ns	ns	ns	**	**										
**Days Tmax ≥ 30°C**	*	**	ns	ns	ns	**	**	ns									

**Powdery mildew treatments**	ns	**	ns	ns	ns	ns	ns	ns	ns							
**Downy mildew treatments**	ns	**	ns	ns	ns	ns	ns	ns	ns	**						
**Disease control treatments**	ns	**	ns	ns	ns	ns	ns	ns	ns	**	**					
**pH**	**	ns	ns	ns	ns	**	**	ns	**	ns	ns	ns				
**Total rainfall**	ns	ns	ns	ns	ns	ns	ns	**	ns	ns	ns	ns	ns			
**Tmax**	ns	**	ns	ns	*	**	**	ns	**	ns	ns	ns	ns	ns		
**Tmin**	ns	**	ns	ns	**	**	**	**	ns	ns	ns	ns	**	ns	ns	
**WI**	**	ns	ns	ns	**	**	**	**	**	ns	ns	ns	**	ns	ns	**

(b)

	TA	Year	TSS	Harvest date	CI	GST	HI	LGS	Days Tmax ≥ 30 °C	pH	Total rainfall	Tmax	Tmin	WI
**TA**	1													
**Year**	0.11	1												
**TSS**	−0.64	0.00	1											
**Harvest date**	0.17	0.08	0.20	1										
**CI**	−0.18	0.38	−0.08	−0.01	1									
**GST**	−0.08	0.25	0.02	0.01	0.41	1								
**IH**	−0.05	0.64	0.08	0.04	0.34	0.78	1							
**LGS**	0.13	−0.13	0.01	−0.06	−0.40	−0.39	−0.24	1						
**Days Tmax ≥ 30°C**	0.05	0.30	0.03	0.03	−0.16	0.64	0.55	−0.05	1					
**pH**	−0.61	−0.15	0.62	−0.07	0.09	−0.02	−0.14	0.11	−0.07	1				
**Total rainfall**	−0.04	0.50	0.02	0.04	0.55	0.00	0.31	−0.48	−0.39	−0.17	1			
**Tmax**	0.16	−0.07	0.01	−0.10	−0.19	0.28	0.13	0.50	0.31	0.11	−0.60	1		
**Tmin**	0.04	0.72	0.05	−0.04	0.31	0.34	0.67	−0.06	0.35	−0.14	0.41	0.22	1	
**WI**	−0.03	0.56	0.07	0.02	0.34	0.84	0.98	−0.20	0.63	−0.12	0.22	0.20	0.63	1

Pairwise two-sided p- values:

	TA	Year	TSS	Harvest date	CI	GST	HI	LGS	Days Tmax ≥ 30 °C	pH	Total rainfall	Tmax	Tmin	WI
**TA**														
**Year**	ns													
**TSS**	**	ns												
**Harvest date**	ns	ns	ns											
**CI**	ns	*	ns	ns										
**GST**	ns	ns	ns	ns	**									
**HI**	ns	**	ns	ns	ns	**								

**LGS**	ns	ns	ns	ns	**	*	ns						
**Days Tmax ≥ 30°C**	ns	ns	ns	ns	ns	**	***	ns					
**pH**	**	ns	**	ns	ns	ns	ns	ns	ns				
**Total rainfall**	ns	**	ns	ns	**	ns	ns	**	ns	ns			
**Tmax**	ns	ns	ns	ns	ns	ns	ns	**	ns	ns	**		
**Tmin**	ns	**	ns	ns	ns	ns	**	ns	ns	ns	**	ns	
**WI**	ns	**	ns	ns	ns	**	**	ns	**	ns	ns	ns	**

Table S4. Precipitation in the hydrological periods (hydrological summer (May–October) and hydrological winter (November–April) for the two study areas and Seleaninov hydrothermic coefficient (HTC) during the growing season (1 April–30 September). (**a**) Western, and (**b**) eastern grape growing area of PDO 'Orvieto' (central of Italy).

(a)

Grape growing area	Year	Total Precipitation (mm)	Hydrologial summer (May–Oct) (mm)	Hydrological winter (Nov–Apr) (mm)	HTC
Western area	2000	636	461	192	1.9
	2001	733	378	469	1.6
	2002	705	348	226	1.9
	2003	726	247	506	0.9
	2004	1079	333	502	1.4
	2005	1126	791	311	1.4
	2006	739	743	316	1.3
	2007	415	326	148	0.9
	2008	1386	592	273	1.7
	2009	886	831	327	2.0
	2010	1217	726	392	1.6
	2011	487	638	186	1.0
	2012	869	206	386	1.0
	2013	1014	818	404	1.5
	2014	1263	654	378	1.7
	2015	951	783	395	1.5

(b)

Grape growing area	Year	Total Precipitation (mm)	Hydrologial summer (May–Oct) (mm)	Hydrological winter (Nov–Apr) (mm)	HTC
Eastern area	2005	1186	619	452	2.3
	2006	378	496	223	1.0
	2007	247	228	113	0.3
	2008	1056	228	230	1.1
	2009	998	824	446	2.2
	2010	1084	577	445	2.1
	2011	594	622	289	1.0
	2012	753	183	295	1.3
	2013	1151	856	466	2.8
	2014	1306	818	404	2.7
	2015	832	659	372	1.9

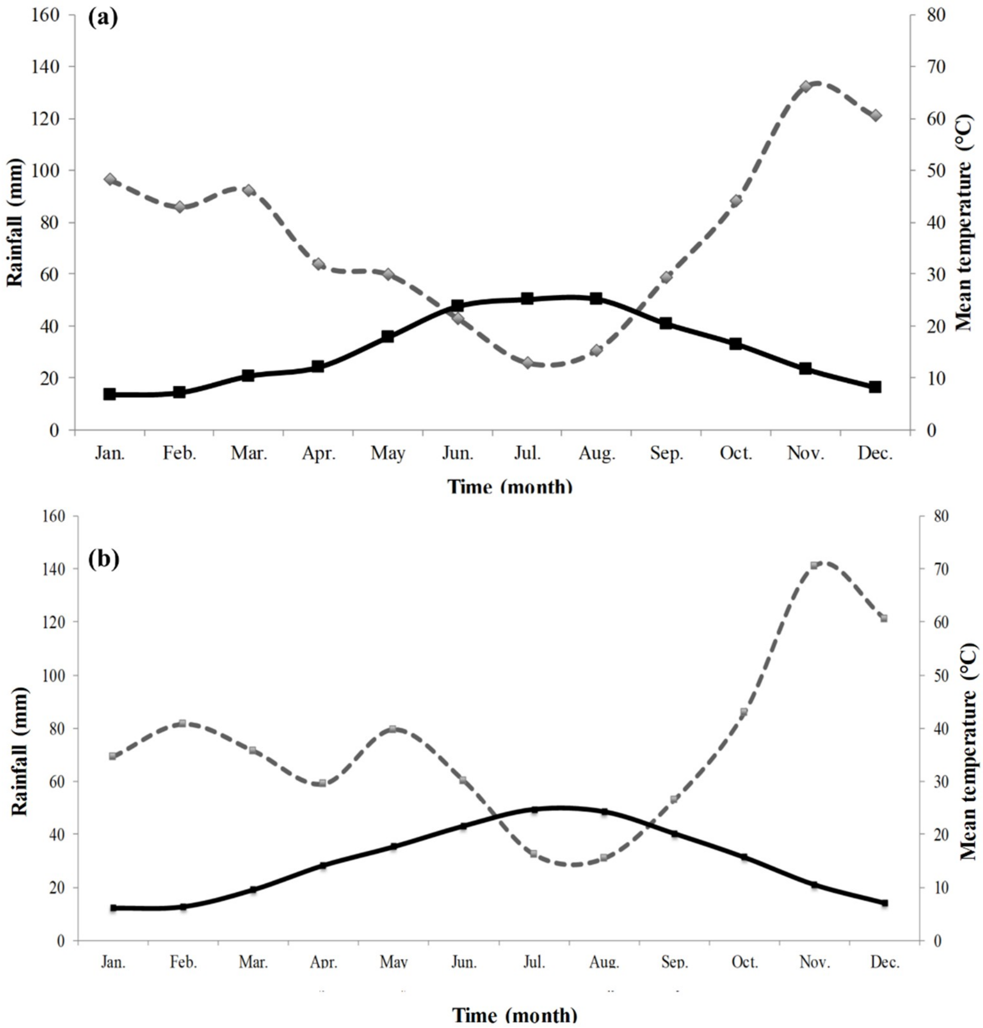

Figure S1. Ombrothermic diagrams of the western sub-area (**a**) and of the eastern sub-area (**b**). The amount of total rainfall. Y axis, left side; mean temperature. Y axis, right side. Data refer to the mean values of 10 year (2005–2015).

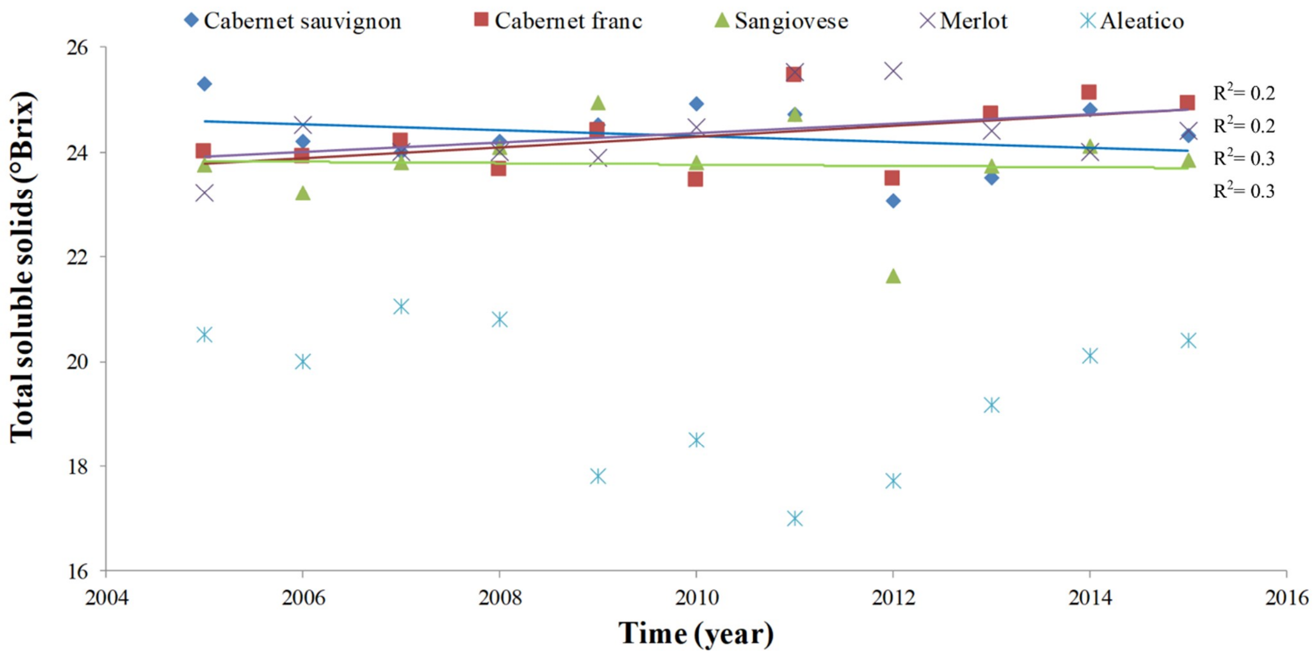

Figure S2. Total soluble solids (°Brix) trends for red varieties related to seasons.

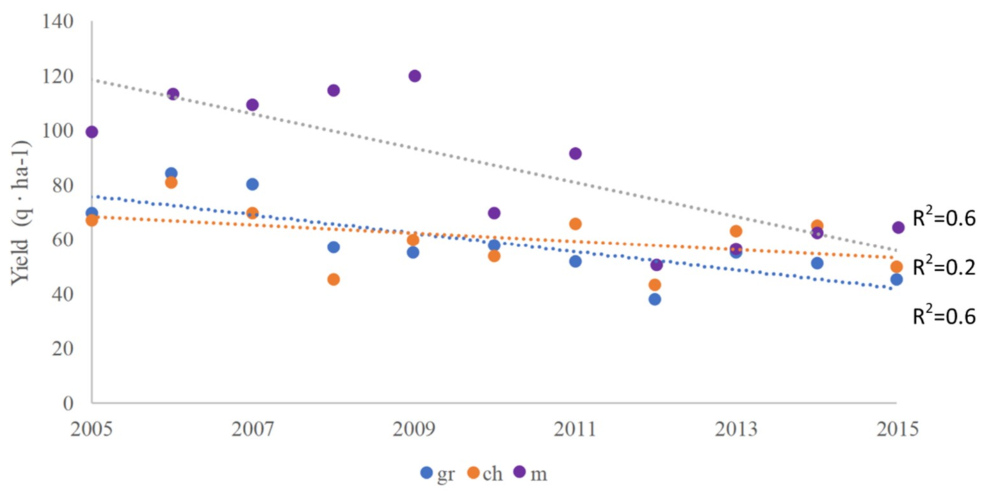

Figure S3. Trend in grapevine yields over the period 2004-2015 for some white and red varieties. CH. Chardonnay; GR. Grechetto; M. Merlot.
